# Military Graveyards and Expitaphs in India—II

**Published:** 1889-03-09

**Authors:** 


*(To be continued.)*

### MILITARY GRAVEYARDS AND EPITAPHS IN INDIA.—II.

BY AN OLD INDIAN.

*(Continued from page 12.)*

OF the epitaphs—"the chisel's slender help to fame"—so common in Indian graveyards, not much can be said in the way of approval, for they consist almost entirely of jingling scraps of ungrammatical doggerel, miserable, though doubtless well meant, parodies of scriptural phrases, pitiful appeals to compassion, incoherent exhortations to virtue, or exaggerated recitals of the merits of the deceased. They are often more calculated to excite ridicule than reverence, and to elicit a smile instead of solemnity. We select a few of the least objectionable, and need scarcely say that our object in doing so is not to sneer at or censure the pious aspirations of surviving relatives, but to show their incongruity, and thereby uggest a modification of the tone or taste in which they are now generally expressed. A disconsolate widow writes over the grave of her husband, a Scotch schoolmaster, at Futtehgurh, the sounding lines of Campbell:

"The spirit shall return to Him
That gave its heavenly spark;
Yet think not, Sun, it shall be dim
When thou thyself art dark.
No, it shall live again and shine
In bliss unknown to beams of thine.
By Him recalled to breath
Who captive led captivity,
Who robbed the grave of victory,
And took the sting from death."

On the headstone of an Irish quartermaster-sergeant at Agra is inscribed the following, which, as it has met with some variations more than once elsewhere, would appear to be a favourite:

"Affliction sore long time he bore,
Physicians were in vain,
Till God was pleased by death to save,
And free him from his pain."

A bereaved husband at Kurnaul bewails the loss of his partner in a strain of hopeful philosophic resignation, which speaks well for his faith, but sounds more like a stave from a drinking song by Dibdin or Pierce Egan than the sober outpouring of genuine grief:

"Here we suffer grief and pain;
Here we meet to part again;
In heaven we part no more.
O! that will be joyful,
Joyful, joyful, joyful,
O! that will be joyful
When we meet to part no more."

As to style, every variety—"from grave to gay, from lively to severe"—is cultivated, and it is not easy to say which predominates.

A father bewails the loss of a child in a strain of farfetched allegory and sorrowful pathos which one would look for in vain in the *Pilgrim's Progress* or the writings of "Satan" Montgomery:

"On life's wild ocean sorrowful and pained,
How many voyagers their course perform:
This little bark a kinder fate obtained,
It reached the harbour ere it met the storm."

A corporal of artillery is made to cry from the grave in the "high falutin" style of our Yankee brethren—the *Siste Viator, heroam Calcas* vein of mediæval Latinity—

"Stop, stop, dear friends, as you pass by,
As now are you so once was I,
As I am now so you will be,
Therefore prepare to follow me."

A third, distrusting his rhyming capacity, or overwhelmed by the poignancy of his grief, writes—

"Time has come when wife, husband
And children had to part,
And not without feelings from
Each other's heart."

